# A Janus‐headed electrolyte and ‘RALES' disease’

**DOI:** 10.1002/ehf2.12173

**Published:** 2017-06-26

**Authors:** Daniel Kraus, Christoph Wanner, Bettina J. Kraus

**Affiliations:** ^1^ Divisions of Nephrology and Cardiology, 1st Department of Medicine, Comprehensive Heart Failure Centre (CHFC) Würzburg University Hospital Würzburg Germany

In ancient Roman mythology, Janus is the god of beginnings and transitions. Janus has two faces, one looking back into the past and one looking ahead into the future (*Figure* 1). According to Ovid, having two faces also enabled him to never turn his back on his adored Cardea.^1^ In the figurative sense, something that is Janus‐headed is ambivalent and must be seen in context. In this regard, potassium is the Janus among electrolytes in heart failure.

**Figure 1:**
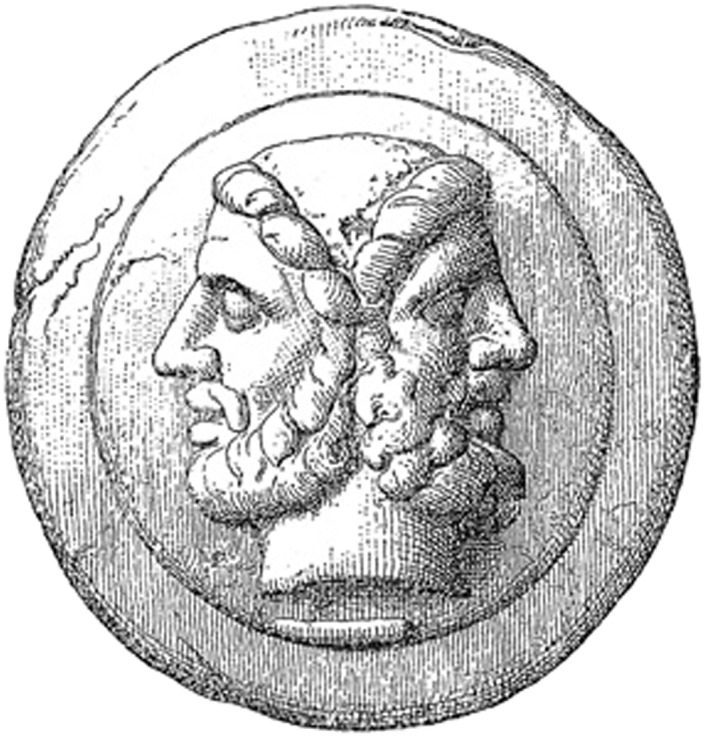
Coin depicting Janus. This image is in the public domain. Source: https://commons.wikimedia.org/w/index.php?curid=34884498

Potassium is abundant in cells and sparse in the extracellular space. This concentration gradient helps to build up the transmembrane potential that is indispensable for electrical signal conduction in the heart and in other organs. A decrease in extracellular potassium concentration augments, and an increase dampens excitability. Both conditions can severely impair normal physiological function.

Serum potassium levels are largely determined by urinary potassium excretion.^2^ By acting on the expression of sodium and potassium transporters in the collecting tubules, the renin–angiotensin–aldosterone system (RAAS) causes sodium retention and potassium excretion.

In patients with heart failure (HF), low levels of serum potassium are associated with increased mortality. Potassium depletion may result from neurohumoral RAAS activation in these patients, as well as from diuretic treatment, a mainstay in the management of heart failure. In the SOLVD trial cohort, the use of non‐potassium‐sparing diuretics was an independent risk factor for sudden cardiac death.^3^

The RALES, EPHESUS, and EMPHASIS‐HF trials have firmly established the role of potassium‐sparing mineralocorticoid receptor antagonists (MRA) in the treatment of heart failure.^4^, ^5^, ^6^ Aldosterone and eplerenone retain potassium and have direct effects on the myocardium. Because aldosterone levels are often increased despite RAAS blockade,^7^ simultaneous application of MRA has an additive effect over ACE inhibitors and angiotensin receptor blockers.

On the other hand, the concomitant use of MRA and other RAAS blocking agents may put patients at risk of fatal arrhythmias due to hyperkalaemia—Janus' second face. This is most commonly seen in those that exhibit reduced aldosterone levels in the first place, e.g. due to hyporeninaemic hypoaldosteronism.^8^ Elderly patients and those with diabetes mellitus or/and kidney disease are at highest risk.

In the wake of the landmark MRA trials, the prescription rates for MRA have sharply increased.^9^, ^10^ A concomitant increase in the rates of hyperkalaemia has been reported in one study (*Figure* 2),^9^ but not in another.^10^ However, the coincidence of hospital admissions due to very severe hyperkalaemia and the dual use of MRA and other RAAS blocking medications is now such a commonplace clinical experience that the term ‘RALES' disease’ (Morbus RALES) has gained currency among emergency physicians.

**Figure 2:**
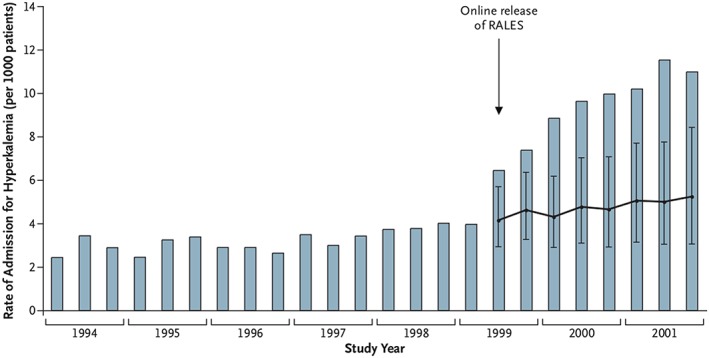
Time course of the rates of hospital admissions for hyperkalaemia before and after the publication of the original RALES trial. Figure taken from Juurlink *et al*.^9^

To assess the actual risk of patients on dual MRA and other RAAS blocking treatment, we queried Würzburg University Hospital's Clinical Data Warehouse (*Table* 1). The Data Warehouse is a clinical registry that includes every inpatient and outpatient case managed by any department at this tertiary care centre. Out of ~5 million cases, we identified 118 691 cases (2.4%) between 2003 and January 2017 with at least one RAAS blocking agent (including an MRA). Dual prescription of an MRA and another RAAS blocking agent increases the risk of ‘RALES' disease’ across all strata of renal function (*Table* 1).

**Table 1:** Incidence of hyperkalaemia among inpatient and outpatient cases between 2003 and January 2017 at Würzburg University Hospital, regardless of medical specialty

Renal function (eGFR)	≥60 mL/min/1.73 m^2^		≥30 and <60 mL/min/1.73 m^2^		<30 mL/min/1.73 m^2^		
MRA or other RAAS blocker	Either	Both	Either	Both	Either	Both	
*n*	72 350	5942	26 408	4339	8709	943	
Age, years (SD)	63.8 (15.4)**	65.5 (13.2)**	74.9 (10.9)**	73.6 (10.4)**	71.6 (14.4)**	74.9 (11.0)**	*
Mean eGFR, mL/min/1.73 m^2^ (SD)	86.6 (19.5)**	81.7 (16.6)**	47.1 (8.4)	46.5 (8.3)	18.3 (7.9)**	21.5 (6.8)**	*
Mild hyperkalaemia, *n* (%)	649 (0.9)**	114 (1.9)**	723 (2.7)**	173 (4.0)**	885 (10.2)	90 (9.5)	*
Severe hyperkalaemia, *n* (%)	227 (0.3)	32 (0.5)	253 (1.0)**	83 (1.9)**	678 (7.8)**	133 (14.1)**	*

eGFR, estimated glomerular filtration rate; MRA, mineralocorticoid receptor antagonist; RAAS, renin–angiotensin–aldosterone system.

Cases are divided according to renal function at presentation (eGFR, calculated with the CKD‐EPI formula) and subdivided into groups with either an MRA prescription or at least one non‐MRA RAAS‐blocking drug, or an MRA in addition to at least one other RAAS blocker. Mild hyperkalaemia, serum potassium level at presentation 5.5 mmol/L or higher, but lower than 6.0 mmol/L. Severe hyperkalaemia, serum potassium level at presentation 6.0 mmol/L or higher.

* *P* < 0.001 for the effects of renal function, medication, and the interaction of both by two‐way ANOVA.

** *P* < 0.001 for the pairwise comparison by Tukey's post‐hoc test within each stratum of renal function.

It is important to realize that a patient's individual risk of developing ‘RALES' disease’ may change over time. It literally changes over time as a patient gets older, because renin and aldosterone levels decline in senescence. Diabetics may gradually develop nephropathy and hyporeninaemic hypoaldosteronism. Acute kidney injury or acute renal failure may occur in any patient. Thus, it is not only important to frequently monitor patients that receive standard‐of‐care treatment for heart failure; it is also paramount to be aware of acute changes in a patient's condition that may precipitate ‘RALES' disease’.

To prevent hyperkalaemia, patients with chronic kidney disease or diabetes mellitus should be counselled to reduce dietary potassium intake (for patients with mildly impaired renal function, there is evidence that potassium supplementation may slow the progression of kidney disease). However, in many cases, dietary restriction will not be sufficient.^11^ Fortunately, there are pharmacological remedies. One approach is to co‐administer potassium binding agents. Sodium polystyrene sulphonate, an ion exchange resin, has been widely used for over 50 years, but its efficacy and safety have been questioned.^11^, ^12^ Patiromer and sodium circonium cyclosilicate (ZS‐9) are two alternative ion exchange agents.

Patiromer effectively prevents ‘RALES' disease’ with normal and reduced renal function.^11^ It binds molecules other than potassium as well and may affect the bioavailability of drugs. Because of this, the United States Food and Drug Administration (FDA) requires a boxed warning, and patients should not take patiromer within 3 hours before or after other medication. This may have implications for patients' adherence to therapy. Patiromer has been approved by the FDA, but not yet by the European Medicines Agency (EMA).

Similar to patiromer, ZS‐9 also effectively lowers potassium levels.^11^ ZS‐9 has been denied approval by the FDA due to manufacturing issues,^13^ but has received a favourable opinion by the EMA's Committee for Medicinal Products for Human Use in February 2017.

Because patiromer and ZS‐9 are novel drugs, there is no experience with long‐term administration of either of them. Patiromer has been studied for up to 12 months and ZS‐9 for up to 4 weeks. Long‐term safety data on frequency and clinical relevance of drug interactions and adverse effects are needed.

Finerenone is a novel, non‐steroidal MRA. Compared with spironolactone and eplerenone, it has higher relative affinity for the heart than for the kidney. In theory, this should allow for better efficacy and lower risk of hyperkalaemia. However, in the recently published ARTS‐HF study, the rates of hyperkalaemia were comparable between the finerenone and eplerenone treatment groups.^14^

Regardless of the approach taken to lower potassium levels, patients at risk for life‐threatening hyperkalaemia must be monitored frequently, especially elderly patients and those with diabetes mellitus and chronic kidney disease. The European Society for Cardiology and the American Heart Association both offer detailed practical guidance.^15^, ^16^ Importantly, acute events such as infection, dehydration, or newly prescribed medications that affect renal blood flow and glomerular perfusion (e.g., non‐steroidal anti‐inflammatory drugs) may precipitate ‘RALES' disease’. Therefore, it is imperative to be alert for such changes in patients at risk.

Taken together, ‘RALES' disease’ is more than a snappy term from the emergency room. It is an admonisher that benefit and risk are the two faces of the same coin, and just like Janus in his courting Cardea, we should never turn our back on our most vulnerable patients.

## Conflict of interest

None declared.
